# The Principles and Practice of Obstetric Medicine and Surgery, in Reference to the Process of Parturition

**Published:** 1845-11

**Authors:** 


Art. IX.—*The Principles and Practice of Obstetric Medicine and Surgery, in reference to the Process of Parturition.*—Illustrated by 148 figures. By Francis H. Ramsbotham, M.D., Fellow of the Royal College of Physicians, &c. &c. &c. A new edition from the enlarged and revised London edition. Philadelphia: Lea & Blanchard. 1845. 8vo. pp. 519.

In size and general appearance this volume corresponds to that of Sir Astley Cooper, noticed in the foregoing article These publishers deserve the thanks of the profession for sending abroad these standard works in a form so pleasing to the eye, and with illustrations which render the text so intelligible. "*The Process of Parturition*" is already well known to most of our readers, and needs, therefore, no commendation from us. The testimonials of the press to its great merits are already so numerous, that no additional praise would be likely to increase its circulation. Physicians who are not in possession of it will find the work at the bookstore of Mr. James Maxwell, through whom we have received an elegant copy of it, together with numerous other volumes, from the publishers.

